# Comparative cell viability of dentin-bonding adhesive systems on human dental pulp stem cells: time-dependent analysis

**DOI:** 10.1186/s12903-024-04438-9

**Published:** 2024-06-07

**Authors:** Magrur Kazak, Ayca Sarialioglu Gungor, Zeynep Ozman, Nazmiye Donmez

**Affiliations:** 1 Department of Restorative Dentistry, Bahcesehir University School of Dental Medicine, Istanbul, Türkiye; 2 School of Medicine and Health Science, Department of Therapeutic Dentistry, BAU International University, Batumi, Georgia; 3 Faculty of Dentistry, Department of Restorative Dentistry, Istanbul Galata University, Istanbul, Türkiye; 4 Faculty of Medicine, Department of Biochemistry, Bezmialem Vakif University, Istanbul, Türkiye; 5 Faculty of Dentistry, Department of Restorative Dentistry, Bolu Abant İzzet Baysal University, Bolu, Türkiye

**Keywords:** Cytotoxicity, Human dental pulp stem cell, Dentin bonding adhesive system, Cell viability, Cell proliferation (WST-1)

## Abstract

**Background:**

Restorative materials are in prolonged contact with living tissues such as oral mucosa, dentin, pulp, periodontal, and periapical tissues. Therefore, the potentially harmful effects of these materials and their components on oral tissues should be evaluated before clinical use. This study aimed to compare the cell viability of different adhesive systems (ASs) on human dental pulp stem cells (hDPSCs).

**Methods:**

Three ASs that combining methacryloyloxydecyl dihydrogen phosphate (MDP) monomer with new hydrophilic amide monomers [Clearfil Universal Bond Quick(CUBQ), Kuraray Noritake], self-reinforcing 3D monomer [Bond Force II(BFII), Tokuyama)], and dual-cure property [Futurabond DC(FBDC), VOCO] were used. Three (*n* = 3) samples were prepared for each group. Dental pulp stem cells were isolated from ten patients’ extracted third molar teeth. Samples were incubated in Dulbecco’s modified Eagle’s medium (DMEM) for 24 h (h), 72 h, and 7 days (d) to obtain extracts. For the control group, cells were cultured without DBA samples. Cell viability of ASs extracts was measured using a cell proliferation detection kit (WST-1, Roche). Statistical analysis was performed using two-way ANOVA and post-hoc (Duncan) tests (*p* < 0.05).

**Results:**

At 24 and 72 h statistically significant differences were determined between control and BFII, control and FBDC groups (*p* < 0.05), while no differences between control and CUBQ groups (*p* > 0.05). On the 7th d, statistically significant differences were found between the control and experimental groups (*p* < 0.05), while no differences between experimental groups (*p* > 0.05). A statistically significant difference was detected for the BFII group over the three-time interval (*p* < 0.05). The lowest cell viability was observed for the FBDC group at 24 h, and the difference was statistically significant when compared with 72 h and 7th d (*p* < 0.05).

**Conclusion:**

All ASs showed different cell viability values at various exposure times. It should be taken into consideration that pH values, as well as the contents of ASs, have a significant effect on the cell viability.

## Background

Restorative materials are in prolonged contact with living tissues such as oral mucosa, dentin, pulp, periodontal, and periapical tissues. Therefore, the potentially harmful effects of these materials and their components on oral tissues should be evaluated before clinical use. The materials should be put into clinical use within the information obtained after evaluation [1].

Biocompatibility tests are divided into three sections: in vitro tests, in vivo (animal experiments), and usage (clinical) tests [2, 3]. Cytotoxicity is the most used test method for in vitro biocompatibility assessments [2]. There are several in vitro test models for screening of biocompatibilities of biomaterials, such as direct, indirect contact tests and extract tests [4]. The extract test method is the most used and reliable in vitro evaluation technique [5]. In this method, cells are not in direct contact with dental materials. Dental materials are kept in a liquid environment, such as a nutrient medium, for a certain period. These liquids contain components released from the materials and can be used in cytotoxicity tests [3, 4]. Thanks to this approach, the effects of the material both in direct contact with the cells and away from the cells can be determined [6, 7]. One of the methods frequently used to determine cytotoxicity is the colorimetric analysis method. The most commonly used tests in this method are WST-1, MTT, and XTT. Different from the other tests, WST-1 determines the number of viable hDPSCs based on their mitochondrial activity [8].

Cell cultures are generally used as a biological system in studies to evaluate the cytotoxicity of dental materials [9, 10]. Cell culture studies have three cell types: primary, diploid, and continuous cells [10]. Primary cell cultures contain cells that have just separated from the original tissue. They express the original physiological state [9, 11]. Stem cells known as primary cells and are defined clonogenic, self-renewing progenitor cells. Based on their origin, there are two main types of stem cells: embryonic stem cells and postnatal stem cells. Several types of postnatal stem cells have been isolated from teeth, including dental pulp stem cells (DPSCs) [12].

Adhesive systems (ASs) serve as intermediary materials that establish a connection between dentin tissue and composite resin. They are designed to facilitate the bonding of dentin tissue to composite resin surfaces, thereby ensuring the retention of dental restorations and preventing issues like microleakage and dentin sensitivity [13]. Throughout the years, dentin adhesive systems have undergone several classifications. These advancements have given rise to various dental adhesive approaches, including etch-and-rinse (E&R) and self-etch (SE) techniques [14].

Tokuyama Bond Force II (Tokuyama, Osaka, Japan) is a one-component, one-coat application, self-etching, light-cured, dental adhesive system which contains a phosphoric acid monomer, 2,2-bis[4-(2-hydroxy-3-methacryloylpropoxy)]-phenyl propane (Bis-GMA), triethyleneglycol dimethacrylate (TEGDMA), and 2-hydroxyethyl methacrylate (HEMA) [15].

Recently, Futurabond DC (Voco, Cuxhaven, Germany) has been introduced as the 8th generation bonding agent of nanofilled dentin adhesive. This adhesive contains a substantial quantity of highly functional nano-sized cross-linking agents integrated with silica particles. A key advantage is its dual-cured nature. This all-in-one adhesive comprises two components, effectively eliminating the necessity for a separate etching step while still achieving adhesive properties comparable to total-etch bonds [16].

In the last few years, some manufacturers introduced universal adhesives with a ‘quick bonding’ concept. This universal adhesive, Clearfil Universal Bond Quick (CUBQ, Kuraray, Tokyo, Japan), is instructed to be used quickly following a ‘no-wait’ concept: apply and light cure without waiting. CUBQ has lower HEMA content, higher purity of the functional monomer 10-Methacryloyloxydecyl dihydrogen phosphate (10-MDP), and the new acrylamide monomer technology to improve the adhesive’s infiltration properties [17].

Since dentin-bonding agents come into close and prolonged contact with the vital dentin tissue, their influence on pulp tissue is of great interest, and they must have good biocompatibility [18]. Biocompatibility is a factor that should be prioritized in restorative materials [19, 20].

The significance and novelty of this study is that isolating dental pulpal stem cells requires a certain protocol and evaluating the biocompatibility of current adhesive systems using the extract method. Regardless of the manufacturers claims, the actual impact of the monomers in the adhesive systems on cellular health should be evaluated. Therefore, this study aimed to evaluate and compare the cell viabilities of three different ASs on human dental pulp stem cells (hDPSCs) at different exposure times. The null hypotheses were: (1) Tested ASs will have the same cell viability values at the same exposure time, (2) The pH of the ASs will affect the cell viability, (3) The cell viability of tested ASs will remain the same as the exposure time changes.

## Methods

### Isolation and culture of hDPSCs

**Fig. 1:**
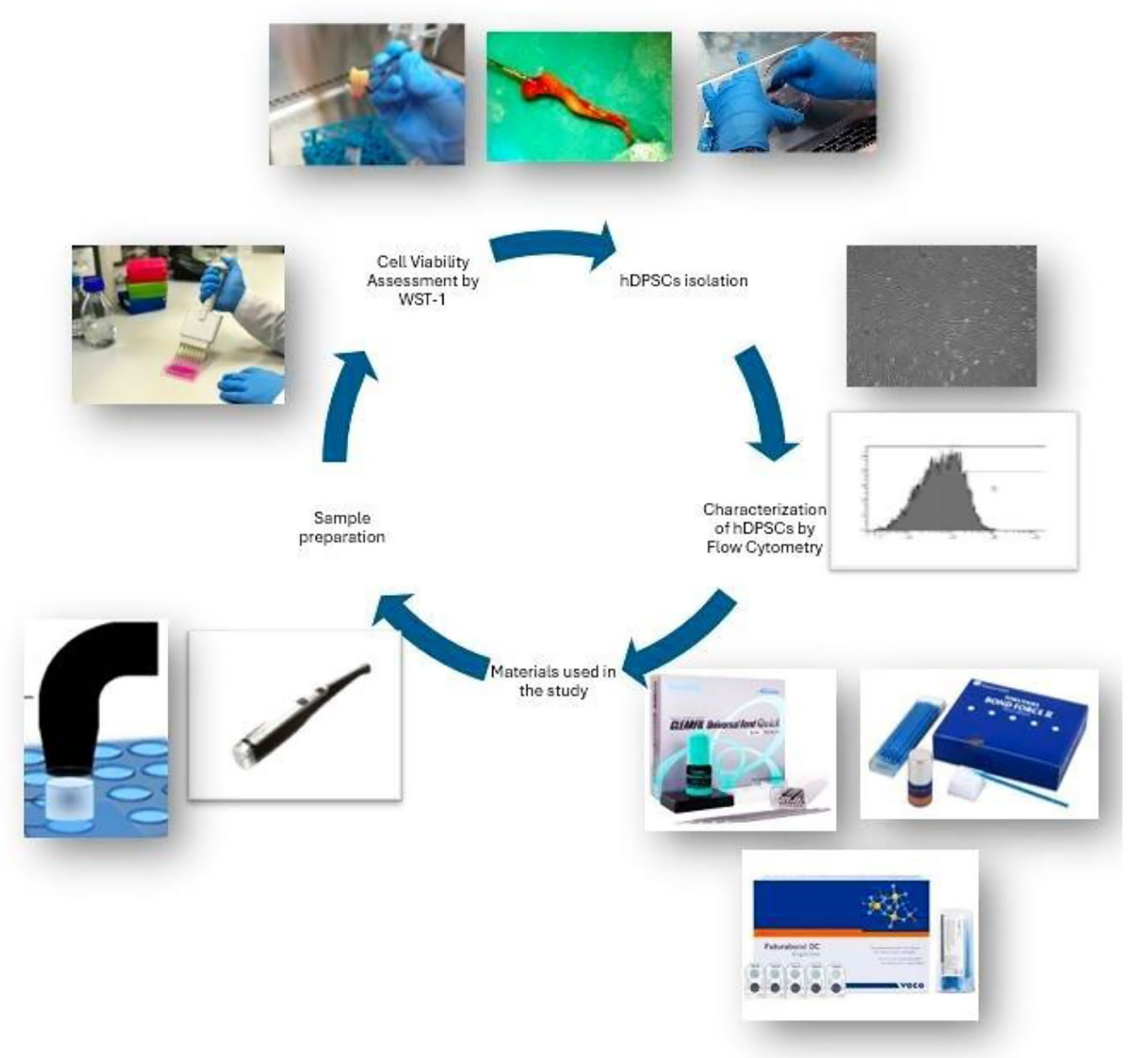
Study design

The study design was shown in Fig. 1.

hDPSCs were isolated from ten patients’ third molar teeth extracted aged 15 to 25. Informed consents were provided from each patient. The informed consent of the minors that participated in the study were obtained from the parents. Following tooth extraction, the molars were submerged in sterile phosphate-buffered saline (PBS; 0.01 mol/L, pH 7.0), and immediately a sterilized diamond fissure bur was used to divide each tooth at the cementum-enamel junction. The pulp tissue from the exposed pulp chamber was gently extracted, minced, and subjected to digestion with 1 mg/mL collagenase/dispase (Sigma-Aldrich, Roche Diagnostics, Mannheim, Germany) for 60 min in a shaking bath at 37 °C.

**Fig. 2:**
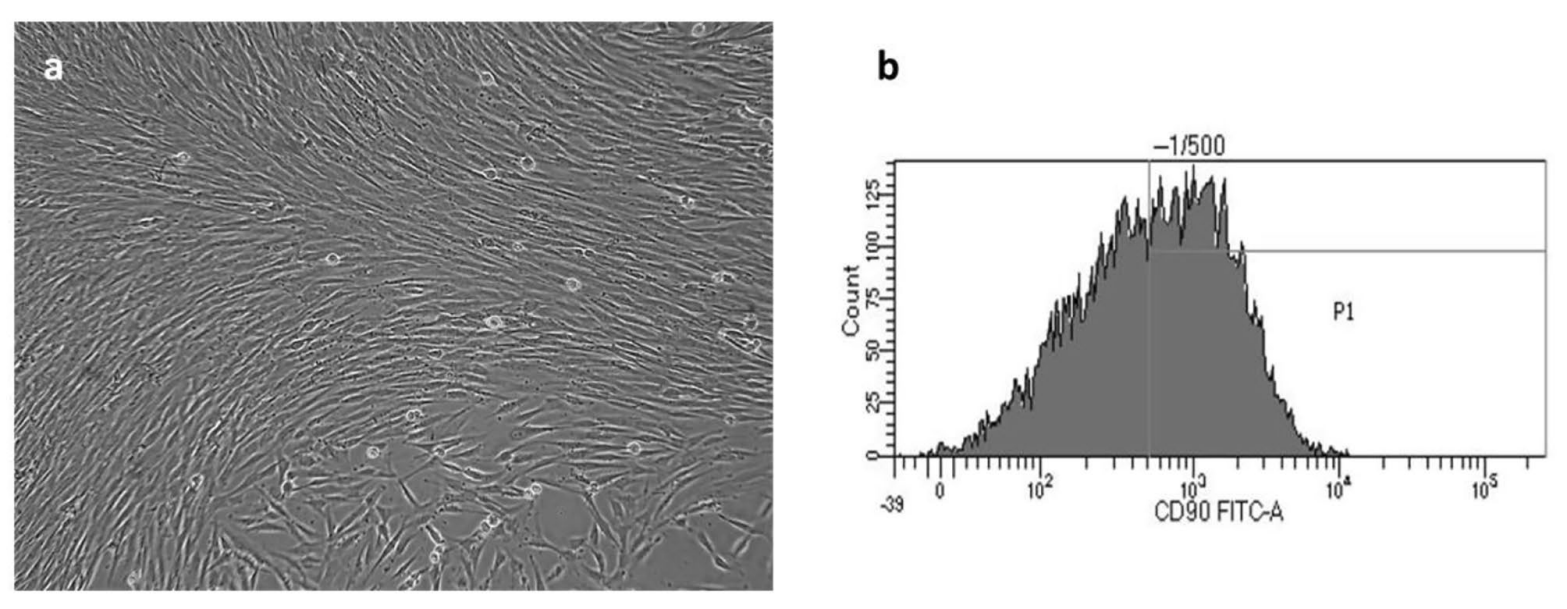
Characterization of the stem cells isolated from human dental pulp tissue. (**a**) Cell morphology was observed under an inverted phase contrast microscope (Nikon TS 100; Tokyo, Japan). (**b**) CD90 surface marker expression by flow cytometry analysis

To maintain cell viability, cells were cultured in Dulbecco’s Modified Eagle Medium (DMEM-F12, Gibco BRL, CA, USA) supplemented with 10% fetal bovine serum (Gibco, CA, USA), 1% l-glutamine, 100 U/ml penicillin, and 100 g/ml streptomycin (Gibco, CA, USA). The cells were incubated at 37 °C in a humidified environment with 5% CO_2_ (Sanyo CO_2_ incubator, Japan). Medium replacement occurred every five days, and cell proliferation was assessed daily under a microscope (Nikon TS 100; Tokyo, Japan). The passage process commenced when the cells covered 70–80% of the culture dish surface. The cells used in this study were obtained from passages 4 through 6. Flow cytometry was employed to assess the surface protein expression of the mesenchymal stem cell marker (CD90) to characterize the cells [21] (Fig. 2).

### Preparation of material extracts

Three ASs, each possessing unique characteristics were used in the study. These included a rapid bonding technology that combines the original MDP monomer with new hydrophilic amide monomers (Clearfil Universal Bond Quick (CUBQ), Kuraray, Tokyo, Japan), a self-reinforcing 3D monomer (Bond Force II (BFII), Tokuyama, Osaka, Japan), and a dual-cure bonding agent (Futurabond DC (FBDC), Voco, Cuxhaven, Germany) (detailed in Table 1). To establish a positive control group, stem cells were cultured without the presence of any DBA samples. The power of the sample size (*n* = 3) was calculated using G*Power software (version 3.1, Heinrich-Heine Dusseldorf University, Dusseldorf, Germany) with a 95% confidence interval, 80% power, and 0.50 effect size values according to one-way analysis of variance (ANOVA)-type power analysis. The minimum sample size was calculated to be 3 specimens per group. Therefore, three DBA samples from each group were applied to cylindrical molds, each with a depth of 2 mm and a diameter of 5 mm, resulting in a total surface area of 1.96 cm^2^. All light-curing procedures were performed using the same curing device (Valo Cordless, Ultradent, South Jordan, USA) for a duration of 10 s which worked in a continuous mode and generated 1,100 mW/cm² of irradiance. It was kept at full charge prior to use, and its irradiance was regularly checked using a dental radiometer (Bluephase Meter II, Ivoclar Vivadent, Schaan, Principality of Liechtenstein). To prevent any contamination, all preparation procedures were meticulously carried out in a sterilized clean bench environment. The prepared materials were then allowed to sit undisturbed at room temperature for 24 h before removing the molds.

The extracts were prepared according to the International Standard Organization (ISO10993-5) protocol [22]. Initially, 0.65 ml of culture medium was introduced to each material and subsequently incubated for 24 h at 37 °C in an environment with a 5% CO_2_ atmosphere. Following this incubation period, the mixture was subjected to centrifugation at 1,000 rpm for a duration of 10 min. The resultant supernatant was then carefully passed through a 0.22-μm filter (ISOLAB Laborgerate GmbH, Eschau, Germany) and securely stored at a temperature of 4 °C. In the final step, the extracts were diluted to a concentration of 5 mg/ml using DMEM (Dulbecco’s Modified Eagle Medium) supplemented with 10% fetal bovine serum. It’s important to note that the same batch of extraction medium was consistently used for each experiment.

**Table 1:** Overview of the materials used in the study

Material and Manufacturer	pH	Serial Number	Composition
Clearfil Universal Bond Quick, Kuraray, Tokyo, Japan	2.3	000036	Bis-GMA, MDP, HEMA, hydrophilic amide monomer, filler, ethanol, water, NaF, photo initiators, accelerator, silane coupling agent, others
Bond Force II, Tokuyama, Osaka, Japan	2.8	124E80	3D-SR phosphate monomer, HEMA, Bis-GMA, TEGDMA water, alcohol, CQ, Catalysts
Futura Bond DC, Voco, Cuxhaven, Germany	1.5	2,111,145	Organic acids, Bis-GMA, HEMA, TMPTMA, CQ, Amines (DABE), BHT, Ethanol, Flourides, Catalysts

Abbreviations: Bis-GMA: Bisphenol A-diglycidyl dimethacrylate, MDP: Methacryloyloxydecyl dihydrogen phosphate, HEMA: Hydroxy Ethyl Methacrylate, NaF: Sodium Fluoride, TEGDMA: Triethylene Glycol Dimethacrylate, CQ: Camphorquinone, TMPTMA: Trimethylolpropane trimethacrylate, BHT: Butylatedhydroxytoluene

### Cell viability assessment

Cell viability assessment followed the guidelines provided by the manufacturer, using a cell proliferation kit (WST-1; Sigma-Aldrich, Roche Diagnostics, Mannheim, Germany). A total of 4,000 hDPSCs were seeded onto 96-well plates, with each well containing 100 mL of culture medium. The cells were then cultured for 24 h (h), 72 h, and 7 days (d) before exposure to various ASs. As a positive control, cells grown in DMEM were used. Following the incubation period, 10 μL of WST-1 solution was introduced into each well, and the cells were incubated for an additional 4 h at 37 °C. Absorbance at 450 nm was measured using a Varioskan Flash Multimode Reader (Thermo Scientific, Waltham, MA, USA). Cell viability was expressed as a percentage relative to the mean of the DMEM controls, which were considered as 100% cell viability. All experiments were conducted in triplicate.

### Statistical analysis

Statistical analyses were conducted using a design of F1-LD-F1 in the nparLD statistical package within the R statistical software (version 4.0.4). Normality of the data was assessed using the Shapiro-Wilk test. Each dataset for cell viability testing was subjected to a two-way ANOVA, followed by post-hoc (Duncan) tests with a significance level set at *p* < 0.05.

## Results

The results of the WST-1 assay of hDPSCs cultured with the extracts of tested materials after 24 h, 72 h, and 7 d of incubation periods are shown in Table 2. Significant differences (*p* < 0.05, *F* = 14.595) were found between the cell viability of control and BFII, control and FBDC groups after 24 and 72-h application of ASs. However, no differences were found between the cell viability of control and CUBQ groups (*p* > 0.05) at the same application times. The lowest cell viability of hDPSCs was determined in the FBDC group at 24 h (7.20 ± 0.34), and the difference was statistically significant when compared with 72 h and 7th day (*p* < 0.05). On the 7th day, statistically significant differences were found between the control and all tested groups (*p* < 0.05), while no differences between the tested ASs (*p* > 0.05).

For the CUBQ group, the cell viability of hDPSCs at the 7th d (22.39 ± 3.80) was significantly lower than 24 h (98.48 ± 8.93) and 72 h (94.26 ± 10.00) (*p* < 0.05). A statistically significant difference was determined for the BFII group over three-time intervals (*p* < 0.05). The cell viability of hDPCs in the CUBQ group was higher than that of other tested AS groups in all time periods (24 h, 72 h, and 7 d) (*p* < 0.05).

The highest cell viability was found in the CUBQ group after 24 h (98.47 ± 8.93), while the lowest cell viability was found in the FBDC at 24 h (7.20 ± 0.34).

**Table 2:** Mean ± Standard error of cell viability values of tested ASs after 24 h, 72 h, and 7 d (*p* < 0.05)

Time	Control	CUBQ	BFII	FBDC
*24 h*	100.00 ± 0.00 ^a^	98.47 ± 8.93 ^Aa^	20.51 ± 2.89 ^Ab^	7.20 ± 0.34 ^Bc^
*72 h*	100.00 ± 0.00 ^a^	94.25 ± 10.00 ^Aa^	14.02 ± 0.58 ^Cb^	16.12 ± 1.05 ^Ab^
*7 d*	100.00 ± 0.00 ^a^	22.39 ± 3.80 ^Bb^	17.16 ± 3.48 ^Bb^	18.35 ± 3.61 ^Ab^

*Different letters within columns and lines indicate statistically significant differences (Uppercases represent columnar differences intragroup, while lowercases represent linear differences intergroup)

## Discussion

The potential cytotoxic effects of ASs with different components and pH values on hDPSC cultures were investigated in the present study. Although there were differences between the cell viability values of the tested ASs at 24 and 72 h, no statistically significant difference was found on the 7th day. Therefore, the first null hypothesis, that the tested ASs will have the same cell viability values at the same exposure time, is rejected.

With each passing day of technological improvement, manufacturers in adhesive dentistry are putting different dental materials with various components on the market. Adhesive systems typically contain a mixture of resin monomers, and it has been shown that these monomers have cytotoxicity and cell-modulating functions [23]. Therefore, due to the presence of new monomer types (such as self-reinforcing 3D monomers or new hydrophilic amid monomers) added to their contents, newly introduced adhesive systems CUBQ and FBII, and FBDC as being a dual-cure adhesive system were included in the study.

Studies conducted in vivo and in vitro have shown that the typical components of adhesive systems, TEGDMA, HEMA, bis-GMA, and 2,4,4-trimethylene diisocyanate (UDMA) monomers, exhibit time- and concentration-dependent cytotoxicity when used in deep cavities or proximity to the pulp tissue [24–26]. Bis-GMA is the most dangerous monomer regarding toxicity levels because it can impair protein synthesis and result in cell death [23]. When compared to HEMA and TEGDMA, bis-GMA is toxic even at low doses, disrupting crucial cell functions such as oxidative stress. Through this action, normal biological cell processes such as cell differentiation, immunological response, and cell repair are indirectly altered. HEMA, a hydrophilic monomer, has a low molecular weight and can penetrate dentin tissue sufficiently to result in permanent pulpal damage. Depending on the dosage, HEMA and TEGDMA may cause necrotic and apoptotic cell death [23, 27]. A study found that Bis-GMA, TEGDMA, and HEMA had more cytotoxic effects on fibroblast cells [28]. The most frequent monomers in ASs, Bis-GMA, TEGDMA, UDMA, and HEMA, were examined by Urcan et al. [29] for their cytotoxic effects. According to their analysis, Bis-GMA was more cytotoxic than TEGDMA, UDMA, and HEMA, respectively. In this study, the CUBQ group, which does not include TEGDMA and has a relatively high pH (2.3), showed the highest cell viability value.

The toxicity of adhesive systems must be evaluated in conjunction with numerous parameters, including viscosity, degree of monomer conversion (% DC), pH, and degree of hydrophilicity [30]. According to ISO 10993-5, cell viability percentages above 80% are regarded as being non-cytotoxic, within 80% and 60% as being mild, 60% and 40% as being moderate, and below 40% as being strongly cytotoxic [31]. In this in vitro study, ethanol-based CUBQ (pH > 2), BFII (pH > 2.5), and FBDC (pH < 2), which contain one or more of the resin monomers, were investigated. Although all tested ASs contained Bis-GMA and HEMA, the FBDC presented the lowest cell viability value at 24 h, this result may be explained by the fact that FBDC has the lowest pH (1.5) compared with the other tested ASs. It has been shown that cells expressing the BAX inhibitor-1 (BI-1) protein exhibit increased cytoplasmic and mitochondrial Ca^+ 2^ ion levels when in contact with an acidic culture medium, which is related to the release of pro-inflammatory cytokines and cell death in a time- and pH-dependent manner [32]. Therefore, the second null hypothesis was accepted.

The cytotoxicity of composite resin and adhesive systems was investigated both immediately and after a 7 d incubation period in the literature [33]. According to the results, all samples were cytotoxic; however, after seven days of incubation, all materials’ cytotoxicity was observed to have decreased. In this study, a decrease was observed in the cell viability of the CUBQ and BFII after 24 h. However, since CUBQ cell viability values are above 80% (at 24 h and 72 h) as a percentage, it can be considered as non-cytotoxic, whereas BFII cell viability values are below 40% at all times tested, so it can be considered as strongly cytotoxic. On the other hand, an increase was observed in the cell viability of FBDC over time but the values were below 40%. Therefore, FBDC can be considered as strongly cytotoxic. This result may be attributed to FBDC being a dual-curing adhesive system. Our results contradict the research by Ulker et al., who used a 3D dentin barrier test to assess the cytotoxicity potential of several commercially available resinous products and found that three out of the four materials under examination significantly reduced cell survival [34]. These low results might be explained by the testing procedure used in the study, as the WST-1 test was carried out indirectly in this study (using material eluates). In contrast, it was carried out directly in their research (using specimens) in the 3D culture system [34]. Therefore, the third null hypothesis that the cell viability of tested ASs will remain the same as the exposure time changes is rejected.

With in vitro tests, it is crucial to consider the type of cell the dental materials come into contact with to determine their cytotoxicity. DPSCs are multipotent cells with a high rate of proliferation, the ability to be safely cryopreserved, and to suppress the immune system; They can be employed confidently in cell cultures to test the cytotoxicity of dental materials. Markers like CD13, CD29, CD44, CD59, CD73, CD90, CD105, and CD146 are expressed by hDPSCs [21]. In this work, we used flow cytometry to identify the hDPSCs and show the expression of the CD90 surface marker.

A practical way for assessing the biocompatibility of biomaterials is the cytotoxicity analysis. Cell viability measurements after exposure to eluted adhesive system components help identify potential toxic effects of such substances [35, 36]. This study’s cellular investigation used the eluates from all tested materials. We acquired eluates of the materials by the International Standard ISO 10993-12 Biological Evaluation of Medical Devices [37], and hDPSCs were treated with various eluates in line with a previously published study [38]. Using the WST-1 assay, the cell viability of the materials under test was evaluated. The 3-(4,5-Dimethylthiazol-2-yl)-2,5-Diphenyltetrazolium bromide (MTT) test and the WST-1 assay are colorimetric methods for assessing cell metabolic activity [39]. Because of the high repeatability and sensitivity properties, as well as higher dynamic ranges than the MTT test, the reliability of WST-1 test is better than the other colorimetric assays [40]. Therefore, in this study the biocompatibility of the ASs was assessed using the WST-1 assay.

Numerous research regarding the time-dependent release of monomers due to inadequate polymerization can be found in the literature. According to some research, it takes 1–7 days to reach full swing [41, 42], while another study recommends waiting six weeks [43]. According to Ratanasathien et al. [44], the toxicity of adhesive systems is significantly influenced by the length of time that active monomers released from adhesive agents. In this study, cell viability assessments were taken at 24 h, 72 h, and 7d to determine the short-medium-long-term cytotoxic effects of the ASs, and it was found that the cytotoxicity changed with the exposure time.

The inability to accurately replicate the oral environment is one of the limitations of this in vitro study. Another limitation is instead of relying on the AS pHs specified by the companies, the pH of the extract samples should be measured. Given the restricted experimental designs, it is challenging to apply the results to clinical situations. The choice of immersion media is a complicated problem. Even when using human saliva, the temperature, chemical, and bacterial factors must be considered to replicate in vivo settings. Furthermore, cytotoxicity was not monitored for durations longer than 7 d. Besides, an evaluation of the degree of conversion of the tested materials, as well as water sorption and solubility should be evaluated. More clinically relevant circumstances need to be the subject of future research.

## Conclusions

It can be concluded that all the ASs evaluated in the study were found to have different cell viabilities on the hDPSCs at various exposure times. Depending on time (24 h, 72 h, 7 d), the cell viabilities of CUBQ and BFII on hDPSCs were decreased, while the cell viabilities of FBDC on hDPSCs was increased. However, CUBQ had better cell viability results, with a significant difference at 24 and 72 h (*p* < 0.05). It should be taken into consideration that pH values, as well as the contents of ASs, have a significant effect on the cell viability.

## Data Availability

The data and materials used for the current study can be obtained by contacting the corresponding author.
